# Supplementary material for: Dynamic perceptive compensation for the rotating snakes illusion with eye tracking
Source: PLoS One. 2021 Mar 4;16(3):e0247937. doi: 10.1371/journal.pone.0247937 (PMC7932078; doi:10.1371/journal.pone.0247937)
Supplement: Attachment — Submitted filename: Response_to_Reviewers.pdf [file pone.0247937.s007.pdf]

Author's response and notes

We have prepared this document in response to the reviewers' and editor's comments and have given some additional notes on our revisions. The text colors used in this document indicate the following:

- Black: Reviewers' and editor's comments that do not require a response
- Red: Reviewers' and editor's comments requiring a response
- Blue: Authors' responses and notes about changes
- Gray highlighting: Authors' revised and added sentences
- **Green bold: Serial numbers of comments and responses**

This document contains the following:

1. Reviewer #1's comments and our responses
2. Reviewer #2's comments and our responses
3. Academic Editor's comments and our responses

Note:
1. The reference numbering has been updated in the revision since we added some reference articles.
2. The manuscript has been revised by an English-language scholarly review service, and the detail comments for the changes in the wording that mean the same content have been omitted in this letter. Correspondent sentences in the manuscript as we mentioned each parts of the comments were underlined and their text color was darken in this document.

1. Reviewer #1's comments

#R1_0---

In a series of three experiments, the authors test whether a gaze contingent display can be used to reduce the perceived motion in the rotating snakes illusion. This is expected to work, given previous research indicating that eye movements increase the strength of the illusion. **(a)**The motivations for this work are a bit unclear in the manuscript. **(b)**Two potential mechanisms are presented in the introduction, but what has been learned from this work in terms of the mechanism of perception of the illusions is not specified in the discussion. **(c)**And I think that the guidance for engineering applications is unclear here, unless the authors simply mean that gaze contingent compensation should be considered a tool that can decrease gaze-related illusions.

Thank you very much for your constructive comments and for the understanding of our research that you show. Based on your comments, we have revised the manuscript in the ways outlined in points (a) to (c) below. We hope that you will examine our responses **#R1_1** to **#R1_11**, which give our detailed responses to your comments on the manuscript.

(a) The motivations of this work

Thank you for your comment.

In this study, we addressed the question whether gaze-dependent illusions, especially rotating snakes illusion (RSI), by using a compensation system including an eye tracker. Thus, we have added the following sentence to the Introduction. A typographical error ("dependes") and wording has also been revised.

- **(Original in ll. 28--30, Introduction)** From these background, our study aims to reduce the effect of the RSI effects that dependes on eye movements. First, we designed and evaluated a dynamic perceptive compensation system and algorithm synchronized with eye movements.
- **(Revised in ll. 29--34)** Our study investigated means of reducing the effects of RSI perception in relation to eye movements. In particular, we addressed the question of whether it is possible to reduce the perception of the RSI in gaze-free conditions through dynamic compensation based on the management of gaze information. First, we designed and evaluated a dynamic perceptive compensation system and an algorithm to be synchronized with eye movements.

We also considered it necessary to describe the motivation for our use application of a dynamic perceptive compensation system, so we have added a description in the Introduction. Please check **#R1_4** for details.

(b) Discussion of two potential mechanisms

Thank you for your comment.

We have added the discussion of the two potential mechanisms of perception of the RSI based on our results, as follows. We also have revised the heading of bullet points in sentence case.

- **(Original in ll. 458--462, Discussion)** Effect of Superposition of Spatial- and Temporal-dependent Compensation: The compensation effectiveness deteriorate for many subjects when spatial- and temporal-dependent compensation are combined. However, the effect is still majorly higher than the still image even in that case. It is necessary to consider space x time interaction for precise reduction of optical illusion because spatial and temporal parameters interact each other.

- **(Revised in ll. 527--546) Effect of superposition of spatially and temporally dependent compensation**: The effectiveness of compensation deteriorates for many subjects when spatially and temporally dependent compensation is combined. It is necessary to consider space x time interactions for the precise reduction of optical illusion, as spatial and temporal parameters interact with each other.
  As described elsewhere, two main mechanisms of RSI related to eye movement have been proposed: refreshing the retinal image through large eye movements and updating the image with small eye movements. Our study dealt only with the former under gaze-free conditions, and the results confirmed that taking large eye movements into account would be effective for compensating for RSI perception. The results also indicated that it would be possible to compensate for RSI perception by physically rotating the image in the opposite direction of the perceptual motion, a conclusion that is consistent with previous studies [12, 14]. On the other hand, the results of Experiment 2 showed that the illusory effect was halved by 500 ms, while larger absolute PSE values were observed in the illusory condition than in the control setting, even at longer compensation time. This suggests that not only to temporally dependent components derive from large eye movements but also that temporally independent components deriving from small eye movements likely exist,

which is consistent with the results of Backus et al. [12] which reported a temporal dependence of the RSI perception in the eye-fixation condition.

(c) Guidance for potential engineering applications

Thank you for your comment.
Perceptive compensation for optical illusions is already in use in engineering applications such as typeface design and icon arrangement. For example, in many typefaces, the intersection of lines in the letter X is intentionally shifted such that the lines are perceived to be connected, exploiting the Poggendorff illusion effect. Taking into account the Hermann grid illusion effect, square icons should not be placed near to each other in a display. In this study, we conducted three experiments on RSI illusions to obtain basic knowledge of how to compensate for gaze-dependent motion illusions.
Following these consideration, we have added guidance for potential engineering applications, i.e., how the findings of our research can be applied to the developments in the field of virtual reality (VR) and human-computer interaction (HCI), which is summarized below.

- **(Original in ll. 500--506, Discussion)** The methods of constructing a perceptual model are broadly divided into a top-down approach that rules visual information processing from a mathematical informatics perspective [4, 5] and a bottom-up approach that generates human perception in a computer using machine learning [26, 35]. Both have succeeded in reproducing and emergent illusions. Both have succeeded in reproducing and emergent illusions. After conducting the above modeling, a couple of individual perceptual parameters are experimentally determined, which realizes to develop more universal compensation system for optical illusions.
- **(Revised in ll. 603--624)** The methods of constructing a perceptual model are broadly divided into a top-down approach that assesses visual information processing from a mathematical perspective [4, 5] and a bottom-up approach that generates human perception in a computer using machine learning[26, 35]. Both have succeeded in reproducing and generating illusions. In the context of our study, it may be possible to construct a model-based illusion compensation system by creating a reproduction model of illusions, including the RSI, for which some parameters can be experimentally determined.
  Finally, we discuss how our proposed methodology, dynamic perceptive compensation synchronized with eye movements, can be used in various fields, such as VR and HCI. The fundamental operations of the measurement of eye movements, image manipulation, and compensation for illusory perceptions in this study can be utilized in relation to gaze-dependent perception. For example, image manipulation by suppressing optical flows that exist in peripheral vision will probably help maintain the sense of balance, which may lead to a reduction in motion sickness. In relation to compensation methodology, the results of our study suggest that it is necessary to account for both spatial and temporal dependence on eye movements. In other words, compensation should be based on the spatio-temporal characteristics of perception in relation to eye movements. Furthermore, our results suggest that it may be necessary to optimize the algorithm parameters using the superposition effect described above. Multi-dimensional optimization methods such as QUEST+ [36] will likely be an important part of effectively improving system optimization.

#R1_1---

The findings are generally sound, but the conclusions are over-stated. In several places, the authors state results as though the optimizations are general to all occurrences of RSI. For example, Experiment 1 is described as finding the "optimal" parameters. Lines 204-206 say that a ~.5 deg/s rotation compensation reduces RSI effects. But this and other values may be specific to the tested individuals and experimental setup (display brightness, specific pattern arrangement, lighting, etc.). These statements should be weakened.
Thank you for your comment.
As you note, the optimal parameters are expected to vary in relation to the lighting environment, display brightness, and specific pattern arrangement. We apologize for the lack of explanation of this point in the manuscript.
First, we have added the following note to remind the audience that the optimal parameter could vary in relation to environment, display brightness, and other factors.

- **(Original in ll. 204--206, Experiment 1)** That is, the compensation against the perceived rotation can reduce the RSI effects when the appropriate angular velocity is chosen: approximately ±0.5 deg./s rotation against perceived motion.

➢ **(Revised in ll. 239--244)** That is, system compensation against the perceived rotation can reduce the RSI effects when the appropriate angular velocity is chosen: forming an approximate ±0.5 deg./s rotation against perceived motion in our experimental design. Note that this optimal parameter is expected to vary in relation to experimental conditions such as the lighting environment, display brightness, and specific pattern arrangement.

Second, in the following two sentences where the specific values of the optimal parameters are mentioned, the text "although the parameter may vary in relation to the environmental conditions, such as display brightness and lighting " is added to weaken the statements to the results of Experiment 1 and the Conclusion. A typographical error ("anglular") and wording have also been also corrected.

➢ **(Original in ll. 226--228, Experiment 1)** Specifically, the compensation anglular velocity of 0.5 deg./s against the perceived motion can reduce the RSI effects in both CW and CCW images in these experiment settings.

➢ **(Revised in ll. 264--268)** Specifically, the compensation angular velocity of 0.5 deg./s against the perceived motion can reduce the effects of the RSI for both the CW and CCW images in these experimental settings, although the parameter may vary in relation to the environmental conditions, such as display brightness and lighting.

➢ **(Original in ll. 518--521, Conclusion)** Preliminary experiment based on paired comparison showed that the compensation by the proposed method reduced the Rotating Snakes Illusion (RSI) effects by setting appropriate parameters; compensation angular velocity at 0.5 deg./s against the perceived motion can reduce the RSI effect.

➢ **(Revised in ll. 635--640)** A preliminary experiment using paired comparison showed that the compensation with the proposed method reduced the effects of the RSI by setting appropriate parameters to our experimental conditions; the compensation angular velocity at 0.5 deg./s against the perceived motion reduced RSI effects, although the optimal parameter could vary in relation to the experimental condition.

#R1_2---

Further, as the authors point out, the effect is dependent on age and probably also a host of other individual factors. Thus, where the authors say the system "can be personalized", it is probably more appropriate to say "must be personalized".

Thank you for your comment.

Previous work has reported that RSI perceptions vary with age and other factors such as image contrast and color. Thus, we agree that the system should be personalized. However, because we did not investigate whether the personalized parameters are essential for applying our compensation system to RSI perception, we have revised the manuscript as follows.

First, it is noted that the compensation parameter should be personalized at the beginning of Experiment 3-1, in the calibration step, where the parameters were actually personalized.

➢ **(Original in ll. 362--363, Experiment 3)** The proportionality constants of each method were determined using the adjustment method for each subject. The subjects observed the stimuli like Fig. 3(a).

➢ **(Revised in ll. 423--427)** Previous studies have reported that the perception of the RSI varies with the individual due to the effects of aging [23], image contrast [15], and other factors. Thus, the compensation parameter should be personalized. In this step, the proportionality constants for each method were determined using the adjustment method for each subject. The subjects observed the stimuli shown in Fig 3(a).

Second, the phrase "can be personalized," found in two places in the manuscript, was changed to "was personalized" to more directly indicate the existence of the calibration step.

➢ **(Original in ll. 405-407, Experiment 3)** Also, by executing the calibration step, the optimal algorithm parameters can be obtained for each individual, and each of the proposed algorithms can be personalized.

➢ **(Revised in ll. 474--475)** Further, through executing a calibration step, the optimal algorithm parameters could be obtained for each individual, and each of the proposed algorithms was personalized.

➢ **(Original in ll. 533-534, Conclusion and future prospects)** Consequently, we realized a system that can control the RSI perception depending on gaze information, and the system can be personalized.

➢ **(Revised in ll. 652--653)** Consequently, we created a system that could control the perception of the RSI depending on gaze information, and this system was personalized.

#R1_3---

The statement that the established parameters "realizes to develop more universal compensation system for optical illusions" (lines 506-7) also seems to overstep the data: this system is specific to RSI.

Thank you for your comment. We apologize for the inadequate description.

Our statement likely confused the application of our compensation system to the reproduction model proposed in previous studies. Thus, we have revised the description of our challenge as follows:

- **(Original in ll. 504 – 507, Discussion)** Both have succeeded in reproducing and emergent illusions.After conducting the above modeling, a couple of individual perceptual parameters are experimentally determined, which realizes to develop more universal compensation system for optical illusions.
- **(Revised in ll. 607--610)** Both have succeeded in reproducing and generating illusions. In the context of our study, it may be possible to construct a model-based illusion compensation system by creating a reproduction model of illusions, including the RSI, for which some parameters can be experimentally determined.

#R1_4---

I don't understand how this study relates to visual perceptual discrepancies in VR and HCI (lines 41-46). If an observer perceives motion when presented with this kind of visual pattern, even though the image is still, this is natural vision. Illusions are perceived in normal vision. Under what VR or HCI situation would you want to augment vision by reducing visual illusions in the periphery—particularly if this process requires a great deal of calibration for specific people and contexts?

Thank you for your comment and we apologize for the ambiguity of our explanation.

Ultimately, our goal was to develop a system that can intervene in human perception by compensating for illusions that depend on eye movements, provide awareness of illusions, and reduce unnecessary illusions. As described in **#R1_0**, perceptive compensation techniques are utilized in various fields, including typeface design, while compensation including gaze information has not yet been investigated. Keeping this in mind, we have added the following description.

- **(Original in ll. 41--46, Introduction)** The technology of controlling gaze-dependent illusion is not limited to understanding the dynamics of visual perception, but is also applicable to eliminating the visual perception discrepancies that are caused by optical illusions for VR and human-computer interaction (HCI). Our research systematically handles gaze measurement, image manipulation, and compensation of illusory image, which can be used as a standard framework to research on optical illusion in the engineering fields.
- **(Revised in ll. 46--57)** The technology of controlling gaze-dependent illusions is not limited to understanding the dynamics of visual perception. It can also be applied to eliminating the discrepancies in visual perception that are caused by optical illusions in the fields of virtual reality (VR) and human-computer interaction (HCI). By intervening in human perception by compensating for gaze-dependent illusions using a head-mounted display or glasses with an eye tracker, it may be possible to produce a system that make one aware of necessary perception and reduces unnecessary illusions. Because the preception of RSI alters with eye movements, use of this technology means we can achieve the fundamental knowledge necessary to intervene in human perception in relation to gaze information. Our research systematically handled gaze measurement, image manipulation, and compensation for an illusory image, and may be usable as a standard framework for research on optical illusion in engineering fields.

#R1_5---

Line 133 says "Both systems were set to drive at 30 Hz" and it is unclear what two systems are referred to here. Were the display and eye tracker set to 30 Hz? Why are 90 Hz and 60 Hz values also reported in the same paragraph? The current text is unclear. A 90 Hz sampling rate is well below the benchmarks achieved by research-grade eyetrackers, and may negatively impact the saccade detection. However, a 30 Hz rate is definitely too low to be valid: previous work has shown that such low-resolution eye tracking fails to reproduce expected saccadic parameters.

Thank you for your comment and we apologize for the ambiguous description in the device and system specifications. In our study, the eye tracker itself was driven at 90 Hz to detect eye position, and the computation time for each processing loop should be kept within approximately 10 ms to drive the whole system at 90 Hz. However, a bottleneck in the system processing caused a delay of more than 10 ms. According to the data from our pilot study (see S1 Dataset), the computational loop from the detection of eye movement to image display took

20.3±1.1 ms (approximately 50 Hz), and the system worked with this delay as a bottleneck when the devices were synchronized.
Further, as you note, the sampling rate for our eye tracker was below the 1000 Hz benchmark that has been achieved by research-grade eye tracker, so we have revised the description of the possible effects as follows.

- **(Original in ll. 128--136, Experimental setting and stimuli)** Experiment 1 used the system that consisted of the eye tracker at 90 Hz (Tobii Eye Tracker 4C), the laptop PC with the keyboard (ThinkPad T440p, Intel(R) Core i-7-4710 MQ 2.50GHz), and the display at 60 Hz (BENQ G2411HD, 1920 x 1080). Experiments 2 and 3 used the system that comprised an eye tracker (Tobii Eye Tracker 4C) driven at 90 Hz, a PC (Dell, Precision 7920 Tower, Intel(R) Xeon(R) Silver 4210 2.20GHz), and the display at 60 Hz (BENQ G2411HD, 1920 x 1080) driven at 60 Hz. Both systems were set to drive at 30 Hz considering the delay in image processing and displaying. Though it is usual for video presentation at 30 Hz, note that the compensation time Δt has error under 66 ms due to refresh rate limits of the whole system.
- **(Revised in ll. 138--161)** Experiment 1 used a system that consisted of an eye tracker at 90 Hz (Tobii Eye Tracker 4C), a laptop PC with a keyboard (ThinkPad T440p, Intel(R) Core i-7-4710 MQ 2.50GHz), and a display at 60 Hz (BENQ G2411HD, 1920 x 1080). Experiments 2 and 3 used the system incorporating an eye tracker (Tobii Eye Tracker 4C) driven at 90 Hz, a desktop PC (Dell, Precision 7920 Tower, Intel(R) Xeon(R) Silver 4210 2.20GHz), and a display at 60 Hz (BENQ G2411HD, 1920 x 1080). In all experiments, the eye tracker itself was driven at 90 Hz to detect the eye position, and the computation time for each processing loop should be kept within approximately 10 ms to drive the whole system at 90 Hz. However, a bottleneck in system processing caused a delay of more than 10 ms. According to data from our pilot study (S1 Dataset), the computational loop from the detection of the eye movements to the image display took 20.3±1.1 ms (approximately 50 Hz), and the system took this delay as a bottleneck when the devices were synchronized. The sampling rate for our eye tracker was below the 1000 Hz benchmark achieved by research-grade eye trackers, and previous studies have found that degradation in the number of detected saccades and inaccurate estimations of saccade duration occurred with eye trackers that have low sampling rates, although accurate measurement of fixation times and points is possible with such trackers [27, 28]. With our eye tracker, it was difficult to follow the saccade process in detail, and the tracker sometimes failed to detect brief saccades, which led to a failure of compensation based on the detection of these brief saccades. On the other hand, our algorithm, as described above, did not require direct measurement of the number or duration of saccades, so it was assumed that the proposed algorithm was able to work adequately through detecting large eye movements and blinks.

We have added the dataset for the pilot study to the Supporting information, as follows.

- **(Revised in ll. 668--669 , Supporting information)** **S1 Dataset. Dataset of eye tracking log data in the pilot study.** Data from a pilot study to investigate the sampling rate of eye tracking, provided in CSV format.

The RSI reduction can be realized at the level of consumer devices, which suggests the possibility of widespread use of our technology at low cost. Thus, we have added the following sentence to the Conclusion.

- **(Original in ll. 535--537, Conclusion and future prospects)** Consequently, we realized a system that can control the RSI perception depending on gaze information, and the system can be personalized. Based on these results, future prospects are described as follows. We adopted the refresh rate at 30 Hz as a usual video rate, while more precise compensation system can realize the improvement of real-time performance.
- **(Revised in ll. 652--656)** Consequently, we created a system that could control the perception of the RSI depending on gaze information, and this system was personalized. The results of our experiments indicated that the effects of the RSI could be diminished even at the speed of consumer devices, which suggests the possibility of widespread use of our technology at a low cost.

#R1_6---
Lines 228-231 suggest that the system compensated regardless of where participants looked on the display, but this was not specifically tested. True, participants were allowed to gaze freely across the screen. However, the authors assessed compensation as an average across trials, not on the basis of where participants were looking during a trial. The compensation could work better or worse in certain regions of the visual field.
Thank you for your constructive feedback, which is helpful for improving the accuracy of our system.
As you note, we consider that the position from which participants observed the display affected the effectiveness of our compensation system. Although we obtained log data (all data are available in S3 Dataset and examples of

gaze heat map obtained in our experiment are shown in S1 Fig to S3 Fig.) of where participants were looking during each trial, the subjects' responses themselves should have been averaged for the trials, which suggests that the RSI as compensated by the system would have been perceived as more stationary on average.
Therefore, first, we have added the following note to experimental methodology in Experiment 1.

- **(Original in ll. 180--181, Experiment 1)** The fixation point of the eyes was not prepared because the experiment measures the effects of eye movements.
- **(Revised in ll. 207--213)** No eye fixation point was prepared, because the experiment measured the effects of the eye movements. It should be noted that that the subjects gave their responses only once per task after observation of the stimuli; the subjects probably responded their perception, which of the two image sets had moved to a great extent, after considering all of their observation during a task (all data are available as S3 Dataset and examples of the gaze heat map obtained in our experiment are shown in S1 Fig to S3 Fig).

Second, we added the sentence "it is possible that this compensation could work more or less easily depending on the viewpoint of the subjects " to the results in Experiment 1, as follows.

- **(Original in ll. 228--231, Experiment 1)** Also, considering the display size used in this study, the system could compensate even if the eyes move freely within a viewing angle range of 45.1 deg. (1500 px) horizontally and 28.0 deg. (900 px) vertically considering the display range of RSI disks.
- **(Revised in ll. 270--277)** Considering the display size used in this study, the system was able to compensate even when the eyes moved freely on average within a viewing angle range of 45.1 deg. (1500 px) horizontally and 28.0 deg. (900 px) vertically taking into consideration the display range of RSI disks, though it is possible that this compensation could work more or less easily depending on the viewpoint of the subjects. For example, in an environment with significantly different display luminance due to non-uniform illumination, the optimal parameters may differ depending on viewpoint, which would cause a dependence of the viewpoint on system effectiveness.

We have also provided the log data for the eye tracker as S3 Dataset and the examples of gaze heatmap obtained in our experiment are shown in S1 Fig through S3 Fig. We have added mentions of these supplemental materials as follows.

- **(Revised in ll. 672--684)**

- **S3 Dataset. Dataset of eye tracking log data in Experiments 1 to 3.** All data obtained in Experiments 1 through 3 are included. All data are available in tabs in the Excel file.

- **S1 Fig. Examples of gaze heat map obtained in our experiments without translucent stimulus image.** Heat maps for one subject (not the same subject across experiments) are shown for (a) Experiment 1, (b) Experiment 2, and (c) Experiment 3.

- **S2 Fig. Examples of gaze heat map obtained in our experiments with translucent stimulus image.** Heat maps for one subject (not the same subject across experiments) with translucent stimulus image are shown for (a) Experiment 1, (b) Experiment 2, and (c) Experiment 3.

- **S3 Fig. Gaze heat map of all subjects obtained in our experiments.** Heat maps for all subjects are shown for (a) Experiment 1, (b) Experiment 2, and (c) Experiment 3.

#R1_7--
Lines 305-307 say that selection bias "probably occurred". The text says "half of the subjects looked at the non-illusory image after the illusory image" but was this tested? This could be assessed using the eye tracking data that was collected. If it was not tested, please specify that where participants looked—and in what sequence—is speculation.
Thank you for your comment and we apologize for the confusion.
In our study, the statement that "half of the subjects looked at the non-illusory image after the illusory image" in a single trial was not tested, but the statement across all tasks was tested, as the counterbalancing was performed by blocks of tasks grouped by a stimulus image.
That is, the selection bias mentioned in the manuscript does not imply that half of the subjects observed the non-illusory (control) image after the illusory image during a single trial; but instead is in reference to entire tasks. Half of the subjects provided their perception under control conditions following the illusory condition because a

counterbalancing experiment was performed for each block of tasks, grouped by the stimulus images; all of the images of the illusory condition were presented after the controls for half of the subjects. Thus, we revised the presentation as follows.
First, the method used for counterbalancing in Experiment 2, where the tasks were grouped by stimulus image, is now described in the experimental procedures.

- **(Original in ll. 269--270, Experiment 2)** All tasks were presented at random for every subject.
- **(Revised in ll. 315--320)** All tasks were presented at random for each subject. Counterbalancing was performed by blocks of tasks grouped by stimulus image. In other words, half of the subjects were randomly presented with a task for each Δt condition for the illusory image, and after all of the tasks with the illusory image were completed, they were randomly presented with a task in the control condition. The other half received the tasks in the reverse order.

Second, we have added the specification that the selection bias mentioned in the manuscript was probably caused by the counterbalance grouping by trial blocks.

- **(Original in ll. 304--307 in Experiment 2)** On the other hand, almost all PSEs of the controlled condition have negative values except the results for Δt = 250 ms, which can be explained by selection bias in the direction of rotation probably occurred: half of the subjects looked at the non-illusory image after the illusory image for which observers perceived clockwise rotation.
- **(Revised in ll. 359--365)** On the other hand, almost all of the PSEs of the control condition had negative values, except for the results forΔt = 250 ms, which could be explained by potential selection bias in the direction of rotation: half of the subjects first worked on the illusory condition task block, and observed an illusory image due to the counterbalancing performed with the blocks of tasks groped by stimulus images. In other words, some subjects continued to observe an illusory image during the first task block, which may have biased their perception of perceived rotation when observing the control image.

#R1_8--

The authors say that an "effect of superposition" may have prevented the combination of spatial and temporal compensation from being effective, but the authors do not describe this superposition effect (beyond describing the data). What mechanisms do you believe are in play here? Also, the text here says "However, the effect is still majorly higher than the still image even in that case" but algorithms O and C did not have a significant difference—and therefore C is not "majorly higher". The combined compensation algorithm does not appear to be effective, and this should be clear in the discussion.
Thank you for your comment. We apologize for our inadequate description of the effects of superposition.
We assumed that this effect may have been due to the insufficient tuning and selection of the compensation parameters in both the calibration and evaluation steps, caused by the fact that the differences between the images with and without compensation could only be recognized for a short time in peripheral vision.
Based on the above, we have added the following sentences to the Discussion.

- **(Original in ll. 463--465, Discussion)** The system and algorithm can be applied to Fraser-Wilcox illusion groups that have a similar pattern as RSI. These illusion groups cause perceived motion in a still image and the direction of motion is determined by arrangement or brightness of the pattern.
- **(Revised in ll. 547--565)** In addition, we discuss the superposition effects observed in our experiment, namely, that the algorithm taking into account both spatial and temporal dependence (Algorithm C) had lower performance than algorithms considering either one or the other (Algorithms A and B). The reason for this may be that the tuning and selection of the compensation parameters were insufficient in both the calibration and the evaluation steps. Algorithm C compensated for this illusion by temporary rotation only to the peripheral vision alone. Thus, the subjects here were sometimes unable to adjust the parameters sufficiently to clearly distinguish the difference between two images. It was confirmed that Algorithms A, B, and C were more effective than Algorithm U, which suggests that taking gaze information into consideration is essential for RSI compensation, and the effects of the superposition of spatial and temporal dependence requires further investigation through the imposition of restrictions on the parameters and observation times.
  Next, the possible application of the proposed system and algorithm is described. The fundamental points in the development of our system can be applied to other dynamic illusions that depend on gaze information. The system and algorithm can be applied to Fraser--Wilcox illusion groups with a similar pattern to that of RSI. These illusion groups cause perceived motion in still images, where the direction of motion is determined by the arrangement or brightness of the pattern.

In addition, as you note, Algorithms O and C did not show a significant difference, so we have deleted the following sentence in the explanation of the effect because we consider previous assertion not to be supported.

- **(Original in ll. 458--462, Discussion)** Effect of Superposition of Spatial- and Temporal-dependent Compensation: The compensation effectiveness deteriorate for many subjects when spatial- and temporal-dependent compensation are combined. However, the effect is still majorly higher than the still image even in that case. It is necessary to consider space x time interaction for precise reduction of optical illusion because spatial and temporal parameters interact each other.
- **(Revised in ll. 527--531)** **Effect of superposition of spatially and temporally dependent compensation**: The effectiveness of compensation deteriorates for many subjects when spatially and temporally dependent compensation is combined. It is necessary to consider space x time interactions for the precise reduction of optical illusion, as spatial and temporal parameters interact with each other.

Minor:

#R1_9--

While the manuscript is sufficiently detailed, the English language should be improved to ensure that an international audience can clearly understand the text. The current phrasing makes comprehension difficult throughout.

Thank you for your comment. We have revised the language of our manuscript for better comprehension by an international audience. The present manuscript has been revised and improved by an English-language scholarly review service.

#R1_10--

The Supporting Information contains subject-level averages, rather than raw data (trial-by-trial responses). As per PLOS data policy, the data points behind means, medians and variance measures should be available.

Thank you for your comment. We have checked PLOS ONE documentation of data-availability policies (https://journals.plos.org/plosone/s/data-availability). The dataset itself was uploaded as a supplemental file, but the title of the file name was not consistent with the documentation, and it was, ultimately, not cited in the manuscript. As you note, we have added the raw data of each subject in the experiments in the same supplementary file. For example, mention of the dataset has been added to the Experiment 1 section, as follows.

- **(Original in ll. 196--198)** Namely, the lower the SR is, the more effectively the system could reduce the RSI effect. The blue line denotes the results for the CW images, the red line the results for the CCW images.
- **(Revised in ll. 231--234)** Lower SRs indicate more effective reduction of the RSI effect by the system. The blue line denotes the results for the CW images, and the red line gives the results for the CCW images. The original data are available in the Experiment 1 Table in the supplemental file (S2 Dataset).

- **(Original in the caption of Fig, 4)** Experimental results. Each value indicates the average results across the subjects. The horizontal axis indicates the compensation angular velocity of $\Delta\theta$, the vertical axis indicates the average of the selection rate (SR) for subjects to choose the compensated image. Namely, the lower the SR is, the more effectively the system could reduce the RSI effect. The blue line denotes the results for the CW images, the red line the results for the CCW images.
- **(Revised)** Results of Experiment 1. Each value indicates results averaged across the subjects. The horizontal axis presents the compensation angular velocity for $\Delta\theta$, and the vertical axis indicates the average of the selection rate (SR) for subjects for choosing the compensated image. Here, the lower the SR, the more effective reduction of the RSI effects by the system. The blue line denotes the results for the CW images, and the red line gives the results for the CCW images. The original data are available in the Experiment 1 Table of the supplemental file (S2 Dataset).

We have also added the section Supporting Information in which the dataset used in the analysis of our study was mentioned.

- **(Revised in ll. 664--665)** **S2 Dataset. Dataset of Experiments 1 to 3.** All data obtained in Experiments 1 through 3 are included. All data are available in the tabs in the Excel file.

#R1_11--

I am interpreting the reported "0.1%" significance levels as p=.001. If this is not correct, please clarify these percentages.

Thank you for your comment. We apologize for the ambiguous description of the levels of the significance. As you correctly surmise, we mentioned 0.1% as $p < .001$. Also, we gave the p-values after Bonferroni correction (i.e., ten times the original value in Experiment 3), and the correspondence has been corrected to $p < 0.01$, where $p < 0.001$ is multiplied by ten. For example, we have revised the text, as follows.

- **(Original in Abstract)** The comparison results confirm that our compensation system considering gaze information reduces the RSI effect more appropriately than the compensation without gaze information at a significant level of 0.1 \% with Bonferroni correction
- **(Revised)** This confirmed that the compensation system that took gaze information into account reduced the RSI effect better than compensation without gaze information at a significance threshold of $p < 0.01$, calculated with Bonferroni correction.

All of the values in Table 2 have been altered to the values after Bonferroni correction, ten times the original values. The p-value for comparison between Algorithms O and C have been set at 1.0, the maximum p-value, because it exceeds 1.0 after Bonferroni correction.

- **(Original in Table 2)**

P-Value	Algorithm O	Algorithm A	Algorithm B	Algorithm C	Algorithm U
O	-	1.8×10^{-12}	2.2×10^{-5}	1.3×10^{-1}	1.4×10^{-26}
A	-	-	8.4×10^{-3}	8.4×10^{-8}	2.8×10^{-23}
B	-	-	-	4.0×10^{-3}	5.2×10^{-23}
C	-	-	-	-	1.3×10^{-26}
U	-	-	-	-	-

- **(Revised)**

**P-Value**	**Algo. O**	**Algo. A**	**Algo. B**	**Algo. C**	**Algo. U**
**O**	-	1.8×10^{-11}	2.2×10^{-4}	1.0	1.4×10^{-25}
**A**	-	-	8.4×10^{-2}	8.4×10^{-7}	2.8×10^{-22}
**B**	-	-	-	4.0×10^{-2}	5.2×10^{-22}
**C**	-	-	-	-	1.3×10^{-24}
**U**	-	-	-	-	-

2. Reviewer #2's comments

Review on Kubota et al. "Dynamic perceptive compensation for the rotating snakes illusion with eye tracking"

Overall evaluation

#R2_0---
The authors present three experiments on perceptual compensation in the rotating snakes illusion, elaborating earlier work by different authors (e.g., Murakami et al. [14], Otero-Millan et al. [16]). Key innovation in the paper is a dynamic compensation system synchronized with eye movements. The overall writing is clear and the research rationale is convincing.
Thank you very much for your constructive comments and the understanding you show of our research. In response to your comments, we have revised the manuscript as follows. Please check our responses from **#R2_1** to **#R2_4**.

General comments
#R2_1---
1. The abstract would probably benefit from reducing length and making it more succinct.
Thank you for this comment. We have modified the abstract and made it more concise.

- **(Original in Abstract)** This study developed a dynamic perceptive compensation system for perceptual manipulation technology based on optical illusions using an eye tracking device that reduces illusion effects depending on eye movement. Using visual stimuli of Rotating Snakes Illusion (RSI), which is one of the gaze-dependent illusion, large eye movements such as saccades and blinks were detected and perceptive compensation was dynamically performed based on an algorithm according to the characteristics of the RSI perception. Our dynamic perceptive compensation in this study considers three properties: spatial dependence, temporal dependence, and individual dependence. We performed several psychophysical experiments to confirm the effectiveness of the proposed system. After preliminary verification and determination of temporal-dependent function for RSI perception, the effect of gaze information on RSI control was investigated. Seven subjects observed the RSI stimuli that were compensated by our algorithms. Five algorithms were compared using the paired comparison. The obtained parameter values for each algorithm indicate the rotation against the perceived motion made appropriate compensation. The comparison results confirm that our compensation system considering gaze information reduces the RSI effect more appropriately than the compensation without gaze information at a significant level of 0.1 \% with Bonferroni correction, while the spatial- and temporal-dependent compensation has a lower score than the other compensation algorithms that may be due to the fact of superposition effects. Some algorithm dependent on the gaze information can be reduced the RSI effects more stably than the still RSI images. Consequently, our system and algorithm can control the RSI perception dependeing on gaze information, and the system can be personalized. Our research systematically handles gaze measurement, image manipulation, and compensation of illusory image, which can be used as a standard framework to research on optical illusion in the engineering fields.
- **(Revised)** This study developed a dynamic perceptive compensation system for the rotating snakes illusion (RSI) with eye tracking. Large eye movements, such as saccades and blinks, were detected with an eye tracker, and perceptive compensation was dynamically performed based on the characteristics of RSI perception. The proposed compensation system considered three properties: spatial dependence, temporal dependence, and individual dependence. Several psychophysical experiments were performed to confirm the effectiveness of the proposed system. After the preliminary verification and determination of the temporal-dependent function for RSI perception, the effects of gaze information on RSI control were investigated. Five algorithms were compared using paired comparison. This confirmed that the compensation system that took gaze information into account reduced the RSI effect better than compensation without gaze information at a significance threshold of $p < 0.01$, calculated with Bonferroni correction. Some algorithms that are dependent on gaze information reduced the RSI effects more stably than still RSI images, whereas spatially and temporally dependent compensation had a lower score than other compensation algorithms based on gaze information. The developed system and algorithm successfully controlled RSI perception in relation to gaze information. This study systematically handled gaze measurement, image manipulation, and compensation of illusory image, and can be utilized as a standard framework for the study of optical illusions in engineering fields.

#R2_2---

2. The eye-tracking device samples data at relatively low rate (90 Hz), which is comparably small when compared to state-of-the-art vision science labs. Could the authors please comment on the potential impact on the results?

Thank you for your comment.

As you note, the sampling rate of our eye tracker was below the 1000 Hz benchmark achieved by research-grade eye trackers. However, RSI reduction can be achieved at the level of consumer devices, which suggests the possibility of widespread use of our technology at a low cost.

First, we have added a description of possible effects due to the low sampling rate of eye tracking, as follows.

- **(Original in ll. 128--136, Experimental setting and stimuli)** Experiment 1 used the system that consisted of the eye tracker at 90 Hz (Tobii Eye Tracker 4C), the laptop PC with the keyboard (ThinkPad T440p, Intel(R) Core i-7-4710 MQ 2.50GHz), and the display at 60 Hz (BENQ G2411HD, 1920 x 1080). Experiments 2 and 3 used the system that comprised an eye tracker (Tobii Eye Tracker 4C) driven at 90 Hz, a PC (Dell, Precision 7920 Tower, Intel(R) Xeon(R) Silver 4210 2.20GHz), and the display at 60 Hz (BENQ G2411HD, 1920 x 1080) driven at 60 Hz. Both systems were set to drive at 30 Hz considering the delay in image processing and displaying. Though it is usual for video presentation at 30 Hz, note that the compensation time Δt has error under 66 ms due to refresh rate limits of the whole system.
- **(Revised in ll. 138--161)** Experiment 1 used a system that consisted of an eye tracker at 90 Hz (Tobii Eye Tracker 4C), a laptop PC with a keyboard (ThinkPad T440p, Intel(R) Core i-7-4710 MQ 2.50GHz), and a display at 60 Hz (BENQ G2411HD, 1920 x 1080). Experiments 2 and 3 used the system incorporating an eye tracker (Tobii Eye Tracker 4C) driven at 90 Hz, a desktop PC (Dell, Precision 7920 Tower, Intel(R) Xeon(R) Silver 4210 2.20GHz), and a display at 60 Hz (BENQ G2411HD, 1920 x 1080). In all experiments, the eye tracker itself was driven at 90 Hz to detect the eye position, and the computation time for each processing loop should be kept within approximately 10 ms to drive the whole system at 90 Hz. However, a bottleneck in system processing caused a delay of more than 10 ms. According to data from our pilot study (S1 Dataset), the computational loop from the detection of the eye movements to the image display took 20.3±1.1 ms (approximately 50 Hz), and the system took this delay as a bottleneck when the devices were synchronized. The sampling rate for our eye tracker was below the 1000 Hz benchmark achieved by research-grade eye trackers, and previous studies have found that degradation in the number of detected saccades and inaccurate estimations of saccade duration occurred with eye trackers that have low sampling rates, although accurate measurement of fixation times and points is possible with such trackers [27, 28]. With our eye tracker, it was difficult to follow the saccade process in detail, and the tracker sometimes failed to detect brief saccades, which led to a failure of compensation based on the detection of these brief saccades. On the other hand, our algorithm, as described above, did not require direct measurement of the number or duration of saccades, so it was assumed that the proposed algorithm was able to work adequately through detecting large eye movements and blinks.

Second, we have added the following sentence to present the potential impact of the use of a consumer device to the Conclusion.

- **(Original in ll. 535--537, Conclusion and future prospects)** Consequently, we realized a system that can control the RSI perception depending on gaze information, and the system can be personalized. Based on these results, future prospects are described as follows. We adopted the refresh rate at 30 Hz as a usual video rate, while more precise compensation system can realize the improvement of real-time performance.
- **(Revised in ll. 652--656)** Consequently, we created a system that could control the perception of the RSI depending on gaze information, and this system was personalized. The results of our experiments indicated that the effects of the RSI could be diminished even at the speed of consumer devices, which suggests the possibility of widespread use of our technology at a low cost.

#R2_3---

3. I understand the purpose of the current work is to propose and analyze dynamic perceptive compensation procedures, however, from impression is that the new results are not adequately discussed in comparison to the earlier findings. Please comment.

Thank you for your comment. We have added a discussion of our results in comparison to previous studies, as follows.

- **(Original in ll. 458--462, Discussion)** Effect of Superposition of Spatial- and Temporal-dependent Compensation: The compensation effectiveness deteriorate for many subjects when spatial- and temporal-dependent compensation are combined. However, the effect is still majorly higher than the still image even in that case. It is necessary to consider space x time interaction for precise reduction of optical illusion because spatial and temporal parameters interact each other.
- **(Revised in ll. 527--546)** **Effect of superposition of spatially and temporally dependent compensation**: The effectiveness of compensation deteriorates for many subjects when spatially and temporally dependent compensation is combined. It is necessary to consider space x time interactions for the precise reduction of optical illusion, as spatial and temporal parameters interact with each other.
  As described elsewhere, two main mechanisms of RSI related to eye movement have been proposed: refreshing the retinal image through large eye movements and updating the image with small eye movements. Our study dealt only with the former under gaze-free conditions, and the results confirmed that taking large eye movements into account would be effective for compensating for RSI perception. The results also indicated that it would be possible to compensate for RSI perception by physically rotating the image in the opposite direction of the perceptual motion, a conclusion that is consistent with previous studies [12, 14]. On the other hand, the results of Experiment 2 showed that the illusory effect was halved by 500 ms, while larger absolute PSE values were observed in the illusory condition than in the control setting, even at longer compensation time. This suggests that not only to temporally dependent components derive from large eye movements but also that temporally independent components deriving from small eye movements likely exist, which is consistent with the results of Backus et al. [12] which reported a temporal dependence of the RSI perception in the eye-fixation condition.

Specific comments and typos

#R2_4--

- l. 2: The first sentence of the main text is unclear. In what sense is it reproduced?
- l. 29: “depends”

Thank you for your comment and for checking the details of our manuscript. We have corrected the two points that you mentioned, as follows. The latter has been changed to a sentence without "depend", based on the advice of an English-language scholarly review service.

- **(Original in l. 2, Introduction)** It is widely known that visual information is reproduced by the processing of our brain.
- **(Revised in ll. 2--3)** It is widely known that physical light that reaches the receptive fields of the retina is processed by the brain to obtain perceptual information. This process often generates differences between the physical and perceived images.

- **(Original in l. 29, Introduction)** From these background, our study aims to reduce the effect of the RSI effects that dependes on eye movements.
- **(Revised in ll. 29--30)** Our study investigated means of reducing the effects of RSI perception in relation to eye movements.

EOF

3. Academic Editor's comment

#E_0---
Thank you for submitting your manuscript to PLOS ONE. After careful consideration, we feel that it has merit but does not fully meet PLOS ONE's publication criteria as it currently stands. Therefore, we invite you to submit a revised version of the manuscript that addresses the points raised during the review process.
Thank you for your response and your sincere communication during the peer review. We have revised our manuscript based on the constructive comments provided by the reviewers and the editors, and this document contains details on the revisions we have added in response for each review comment. We would appreciate it if you could take a moment to check them.

Please submit your revised manuscript by Jan 23 2021 11:59PM. If you will need more time than this to complete your revisions, please reply to this message or contact the journal office at plosone@plos.org. When you're ready to submit your revision, log on to https://www.editorialmanager.com/pone/ and select the 'Submissions Needing Revision' folder to locate your manuscript file.

Please include the following items when submitting your revised manuscript:

- A rebuttal letter that responds to each point raised by the academic editor and reviewer(s). You should upload this letter as a separate file labeled 'Response to Reviewers'.
- A marked-up copy of your manuscript that highlights changes made to the original version. You should upload this as a separate file labeled 'Revised Manuscript with Track Changes'.
- An unmarked version of your revised paper without tracked changes. You should upload this as a separate file labeled 'Manuscript'.

If you would like to make changes to your financial disclosure, please include your updated statement in your cover letter. Guidelines for resubmitting your figure files are available below the reviewer comments at the end of this letter.

If applicable, we recommend that you deposit your laboratory protocols in protocols.io to enhance the reproducibility of your results. Protocols.io assigns your protocol its own identifier (DOI) so that it can be cited independently in the future. For instructions see: http://journals.plos.org/plosone/s/submission-guidelines#loc-laboratory-protocols

We look forward to receiving your revised manuscript.

Kind regards,

Susana Martinez-Conde
Academic Editor
PLOS ONE

Journal Requirements:
When submitting your revision, we need you to address these additional requirements.

#E_1---
1. Please ensure that your manuscript meets PLOS ONE's style requirements, including those for file naming. The PLOS ONE style templates can be found at
https://journals.plos.org/plosone/s/file?id=wjVg/PLOSOne_formatting_sample_main_body.pdf and
https://journals.plos.org/plosone/s/file?id=ba62/PLOSOne_formatting_sample_title_authors_affiliations.pdf
Thank you for your comment. We checked PLOS ONE style templates you mentioned.
We have revised each of the following items to meet the style requirements.

(a) The heading of the title has been changed to sentence case.

➢ (Original in Title) Dynamic Perceptive Compensation for the Rotating Snakes Illusion with Eye Tracking
➢ (Revised) Dynamic perceptive compensation for the Rotating Snakes Illusion with eye tracking

(b) The author affiliations and corresponding author information have been revised according to the requirements.

➢ **(Original in Affiliations)** Graduate School of Information Science and Technology, the University of Tokyo, Hongo 7-3-1, Bunkyo-ku, Tokyo, Japan
➢ **(Revised)** Graduate School of Information Science and Technology, the University of Tokyo, Bunkyo-ku, Tokyo, Japan

➢ **(Original in Authors)** * yuki_kubota@ipc.i.u-tokyo.ac.jp
➢ (Revised) * Corresponding author
E-mail: yuki_kubota@ipc.i.u-tokyo.ac.jp (YK)

(c) The citation of figures in the manuscript has been revised, as in the following example.

➢ **(Original in ll. 51-52, Related work)** Rotating Snakes Illusion (RSI) shown in Fig. 1 perceives an illusory rotation in a still image [11], which is based on Fraser Wilcox Illusion [3, 18].
➢ **(Revised in ll, 59--60)** The RSI shown in Fig 1 presents an illusory rotation in a still image [11], based on the Fraser--Wilcox illusion [3, 18].

(d) The titles of the captions of figures and tables have been given in bold type, as follows.

➢ **(Original in Fig 1)** Fig 1. Stimulus patterns that perceive the Rotating Snake Illusion (RSI). This illusion comprises four gray-scale patterns: black, light gray, white, and dark gray. The direction of motion changes depending on the pattern arrangement. Our study arranges the patterns repeatedly to enhance the intensity of the illusion.
➢ **(Revised in Fig.1)** Fig 1. **Stimulus patterns that perceive the RSI.** This illusion incorporates four gray-scale patterns: black, light gray, white, and dark gray. The direction of motion changes depending on the arrangement of the pattern. Our study repeatedly aligned the patterns to enhance the intensity of the illusion.

(e) Table 1 has been moved to be placed soon after the spot in the text where it is cited.

(f) The indexes of Tables 1 and 2 have been given bold type, as follows. The actual manuscript uses a LaTeX table environment.

➢ **(Revised in Table 1)**

**Algorithm**	**Ratio of larger scores**	**First quartile**	**Median**	**Third quartile**
O	-	−0.307	−0.043	0.120
A	6 / 6	0.117	0.400	0.780
B	5 / 6	0.153	0.269	0.400
C	4 / 7	−0.164	0.122	0.246
U	1 / 5	−1.316	−0.854	−0.536

➢ **(Revised in Table 2)**

**P-Value**	**Algo. O**	**Algo. A**	**Algo. B**	**Algo. C**	**Algo. U**
**O**	-	1.8×10^{-11}	2.2×10^{-4}	1.0	1.4×10^{-25}
**A**	-	-	8.4×10^{-2}	8.4×10^{-7}	2.8×10^{-22}
**B**	-	-	-	4.0×10^{-2}	5.2×10^{-22}
**C**	-	-	-	-	1.3×10^{-24}
**U**	-	-	-	-	-

#E_2--
2. Thank you for stating the following financial disclosure:
"The funders had no role in study design, data collection and analysis, decision to publish, or preparation of the manuscript."
At this time, please address the following queries:

a. Please clarify the sources of funding (financial or material support) for your study. List the grants or organizations that supported your study, including funding received from your institution.

b. State what role the funders took in the study. If the funders had no role in your study, please state: "The funders had no role in study design, data collection and analysis, decision to publish, or preparation of the manuscript."

c. If any authors received a salary from any of your funders, please state which authors and which funders.

d. If you did not receive any funding for this study, please state: "The authors received no specific funding for this work."

Please include your amended statements within your cover letter; we will change the online submission form on your behalf.

Thank you for your comments. Our research had no support or funding to report. For this reason, we have changed the reporting of our financial disclosures in our cover letter as follows.

- The authors received no specific funding for this work.

**********************Following two responses was added on Jan 26 2021****************************

PONE-D-20-21363R1
Dynamic perceptive compensation for the rotating snakes illusion with eye tracking
Mr. Yuki Kubota

Dear Mr. Kubota,

Thank you for submitting your manuscript entitled "Dynamic perceptive compensation for the rotating snakes illusion with eye tracking" to PLOS ONE. Your manuscript files have been checked in-house but before we can proceed we need you to address the following issues:

#E_3---

1) We note that your manuscript is not formatted using one of PLOS ONE's accepted file types. Please reattach your manuscript as one of the following file types: .doc, .docx, .rtf, or .tex (accompanied by a .pdf).
If your submission was prepared in LaTex, please submit your manuscript file in PDF format and attach your .tex file as "other."
Thank you for your comments and we apologize for forgetting to attach the .tex file.
We have added two LaTeX files to our submission.

#E_4---

2) Your ethics statement should only appear in the Methods section of your manuscript. If your ethics statement is written in any section besides the Methods, please move it to the Methods section and delete it from any other section. Please ensure that your ethics statement is included in your manuscript, as the ethics statement entered into the online submission form will not be published alongside your manuscript.
Thank you for your comments.
We have moved the ethics statement to the Methods section (called "Experimental procedures and conditions" in our manuscript) of each of the three experiments. We hope you will examine the lines ll. 225--227, 325--327, 458--460 in the modified manuscript.
